# Supplementary figures and images for: P-Glycoprotein Inhibitors Differently Affect Toxoplasma gondii, Neospora caninum and Besnoitia besnoiti Proliferation in Bovine Primary Endothelial Cells
Source: Pathogens. 2021 Mar 25;10(4):395. doi: 10.3390/pathogens10040395 (PMC8065907; doi:10.3390/pathogens10040395)

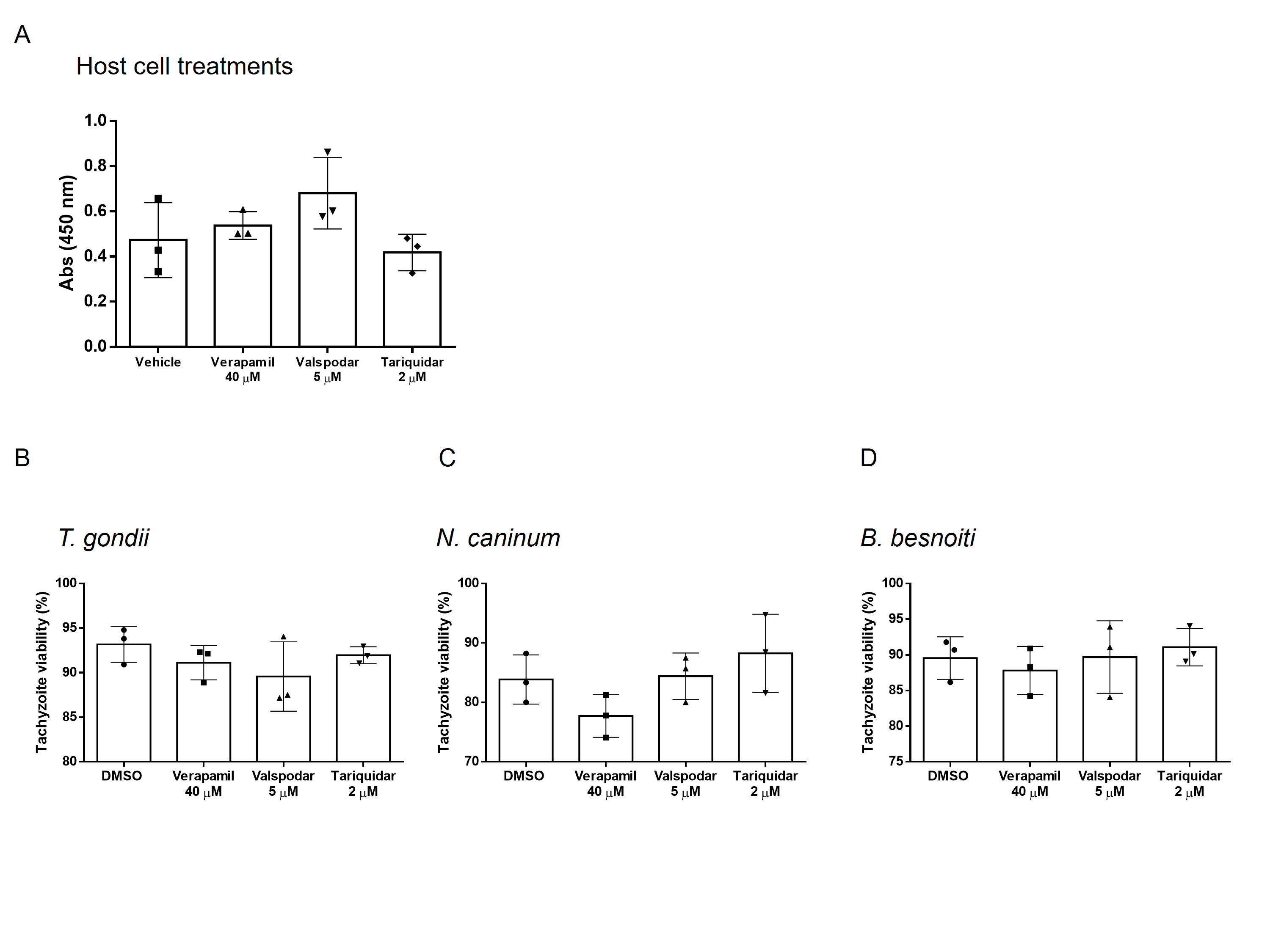

Supplement: Supplementary file 1 [file pathogens-10-00395-s001.zip › Figure S1.tif]

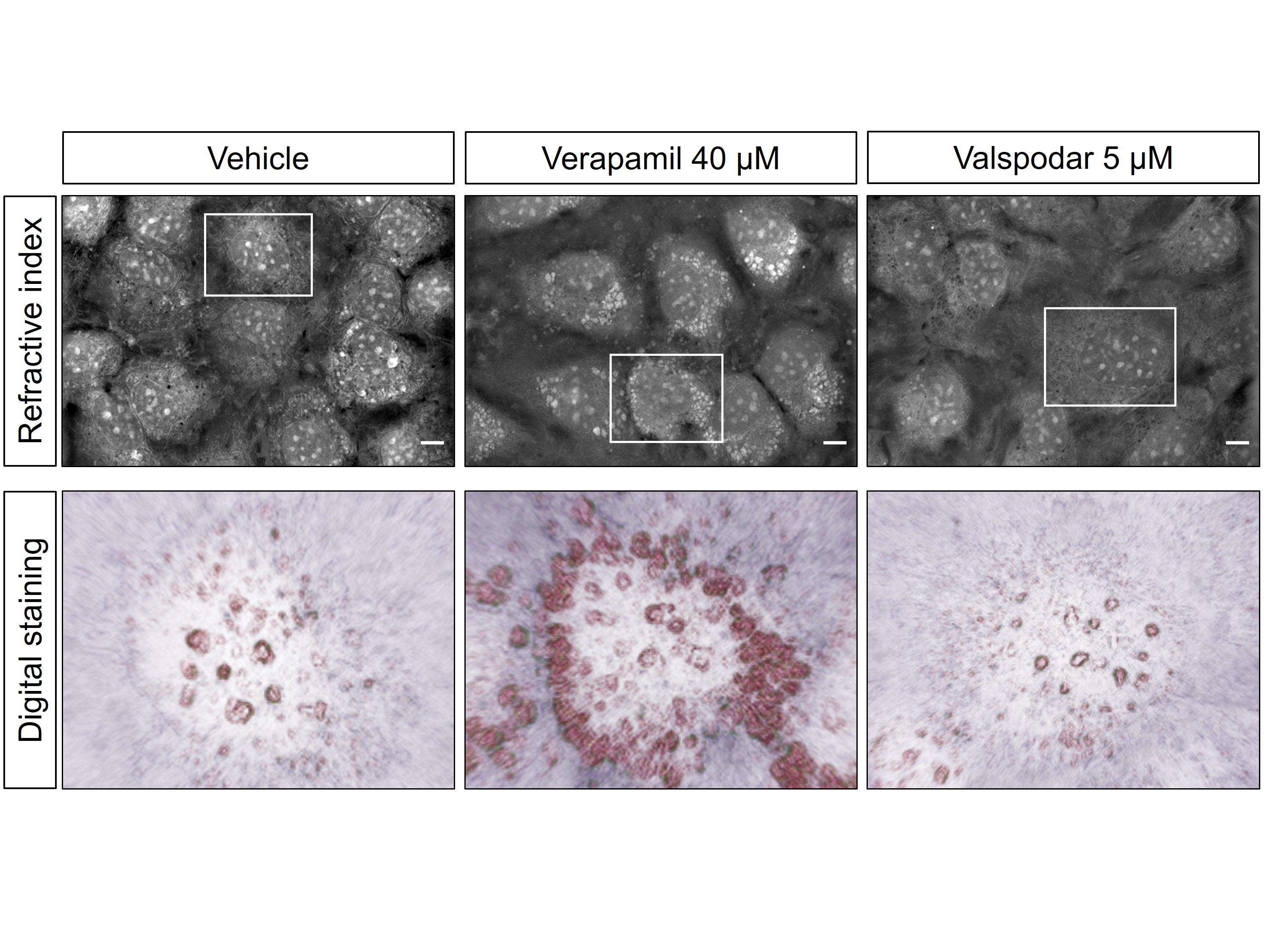

Supplement: Supplementary file 1 [file pathogens-10-00395-s001.zip › Figure S2.tif]

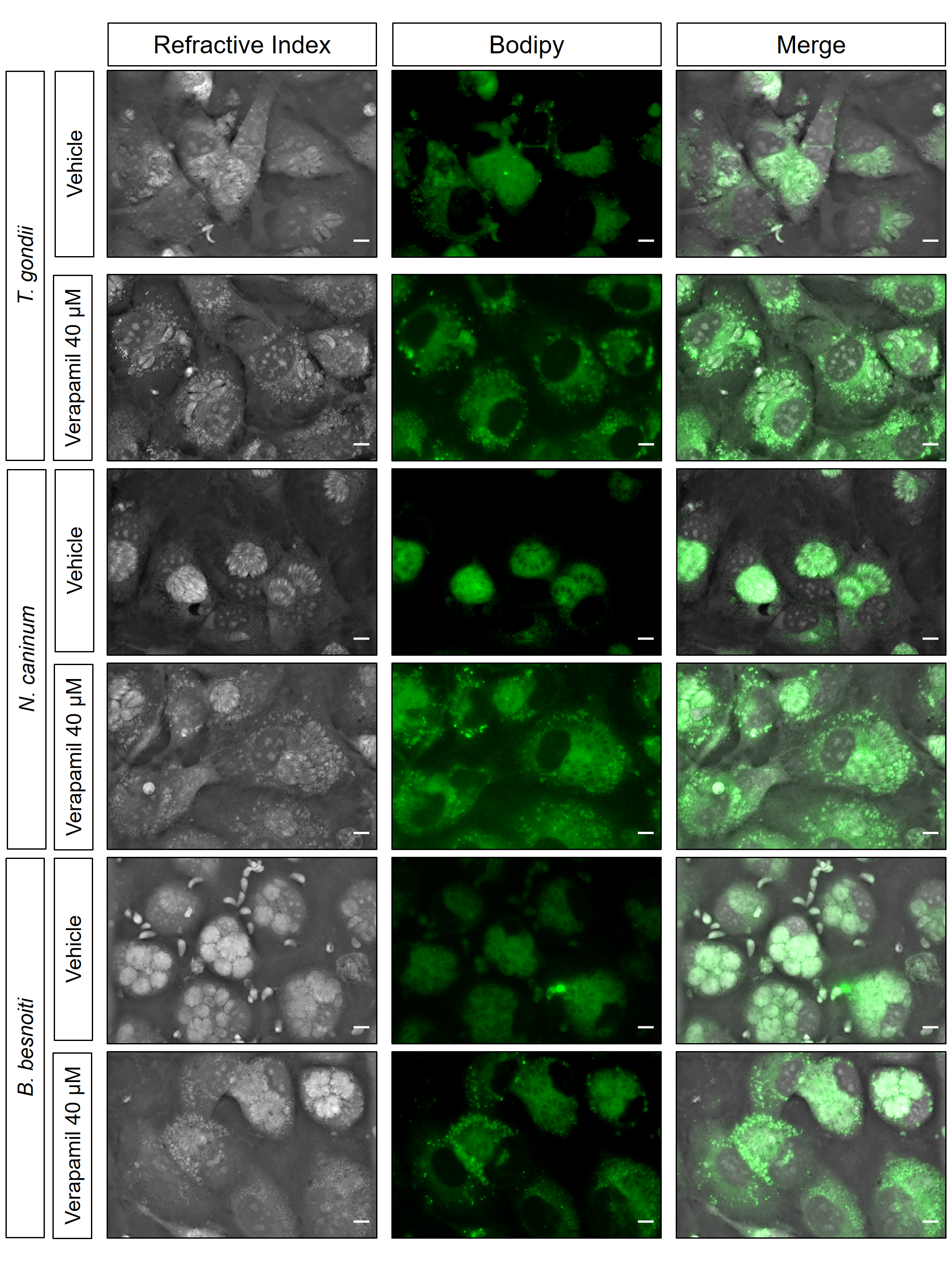

Supplement: Supplementary file 1 [file pathogens-10-00395-s001.zip › Figure S3.tif]
